# Freshwater plastisphere: a review on biodiversity, risks, and biodegradation potential with implications for the aquatic ecosystem health

**DOI:** 10.3389/fmicb.2024.1395401

**Published:** 2024-04-18

**Authors:** Valerio Bocci, Silvia Galafassi, Caterina Levantesi, Simona Crognale, Stefano Amalfitano, Roberta Congestri, Bruna Matturro, Simona Rossetti, Francesca Di Pippo

**Affiliations:** ^1^Water Research Institute, CNR-IRSA, National Research Council, Rome, Italy; ^2^PhD Program in Evolutionary Biology and Ecology, Department of Biology, University of Rome “Tor Vergata”, Rome, Italy; ^3^Water Research Institute, CNR-IRSA, National Research Council, Verbania, Italy; ^4^NBFC, National Biodiversity Future Center, Palermo, Italy; ^5^Laboratory of Biology of Algae, Department of Biology, University of Rome “Tor Vergata”, Rome, Italy

**Keywords:** freshwater plastisphere, biodiversity, antibiotic resistance genes, pathogenic bacteria, plastic biodegradation

## Abstract

The plastisphere, a unique microbial biofilm community colonizing plastic debris and microplastics (MPs) in aquatic environments, has attracted increasing attention owing to its ecological and public health implications. This review consolidates current state of knowledge on freshwater plastisphere, focussing on its biodiversity, community assembly, and interactions with environmental factors. Current biomolecular approaches revealed a variety of prokaryotic and eukaryotic taxa associated with plastic surfaces. Despite their ecological importance, the presence of potentially pathogenic bacteria and mobile genetic elements (i.e., antibiotic resistance genes) raises concerns for ecosystem and human health. However, the extent of these risks and their implications remain unclear. Advanced sequencing technologies are promising for elucidating the functions of plastisphere, particularly in plastic biodegradation processes. Overall, this review emphasizes the need for comprehensive studies to understand plastisphere dynamics in freshwater and to support effective management strategies to mitigate the impact of plastic pollution on freshwater resources.

## 1 Introduction

The term “plastisphere” was coined to describe the unique community of aquatic microbes that colonize plastic debris in marine environments (Zettler et al., 2013). Early studies on marine plastisphere were mainly based on the morphological identification of microorganisms using scanning electron microscopy (SEM), which highlighted the presence of filamentous bacteria and phototrophic eukaryotes (e.g., diatoms) (Carson et al., 2013; Zettler et al., 2013; Oberbeckmann et al., 2014; Amaral-Zettler et al., 2020). The use of biomolecular methods is contributing to improve our understanding of plastisphere biodiversity in freshwater environments revealing the full breadth and complexity of plastic-associated biofilms (Besemer et al., 2012; Besemer, 2015; Di Pippo et al., 2020).

Recent studies have shown that plastisphere is composed of microbial photoautotrophs, heterotrophs, protistan grazers and decomposers, most of which are known as biofilm formers and biofilm-associated microbes (Du et al., 2022; Li W. et al., 2023; Nikolopoulou et al., 2023). Once in water, the plastic debris provides hard surfaces for rapid microbial colonization and a new pelagic habitat for benthonic species (Yang Y. et al., 2020; Dąbrowska, 2021; Haram et al., 2021). Plastics and microplastics (MPs) were reported either as dispersal vehicles for microorganisms of health concern (Di Pippo et al., 2022; Du et al., 2022; Rubin and Zucker, 2022) or as hotspots for horizontal gene transfer, including antibiotic resistance genes (ARGs) (Luo et al., 2023; Chen et al., 2024; Li K. et al., 2024). Furthermore, plastisphere microorganisms were also found to be directly involved in polymer biodegradation (Du et al., 2022; Mishra et al., 2022; Yuan et al., 2022; Li K. et al., 2023).

Much of the current literature has so far demonstrated that several environmental conditions and local factors influence microbial communities developing on plastic surfaces (Hoellein et al., 2014; Oberbeckmann et al., 2018; Amaral-Zettler et al., 2020; Coons et al., 2021; Amaneesh et al., 2023). However, fundamental questions remain unanswered. The role of the polymer type and properties on plastisphere structure and biodiversity is still unclear (Sooriyakumar et al., 2022; Li K. et al., 2023; Miao et al., 2023). In addition, there is a knowledge gap about community assembly over time and on the microbial taxa involved in the different stages of plastisphere succession (Pollet et al., 2018; Amaral-Zettler et al., 2020; Eronen-Rasimus et al., 2022; Forero-López et al., 2022; Wallbank et al., 2022; Miao et al., 2023).

Despite the growing recognition of plastic waste pervasiveness in marine ecosystems and the large body of research focusing on plastic- and microplastic-associated biofilms, the freshwater plastisphere remains relatively understudied. Given the critical importance of quality freshwaters to provide essential services to human health and society, this disparity highlights the need for a more comprehensive understanding of plastic pollution in freshwater ecosystems, which can harbor a complex and diverse array of microorganisms differently sensitive to environmental and anthropogenic pollution (Hoellein et al., 2017; Di Pippo et al., 2020, 2022; Eronen-Rasimus et al., 2022; Nguyen et al., 2023).

This study was entailed to synthetically overview the current knowledge of freshwater plastisphere and the main environmental factors potentially influencing its development and microbial community assembly. In particular, we examined the current understanding of how the plastisphere might affect freshwater ecosystems and human health. We also examined its potential for biodegradation and identified critical aspects that require further investigation.

## 2 Plastisphere biodiversity, taxon composition, and factors affecting microbial community assembly

Once dispersed in water, plastics and MPs are rapidly colonized by planktonic microorganisms, which can adhere and grow onto solid surfaces forming complex plastic-associated biofilms whose biodiversity profiles consistently differ from those of the surrounding environments (see Table 1). While there is a shared consensus on the definition of a new plastic-associated micro-ecosystem with distinct microbiota, it is still debated whether the freshwater plastisphere harbors higher or lower biodiversity than planktonic communities and biofilms formed on natural substrates (McCormick et al., 2014, 2016; Hoellein et al., 2017; Arias-Andres et al., 2018; Wu et al., 2019; Wang et al., 2020; Xue et al., 2020; Galafassi et al., 2021; Kelly et al., 2021).

**Table 1:** Main groups of microorganisms found in freshwater plastisphere.

Freshwater source	Target genes sequenced	Plastics/MPs	Main taxa (database used for taxonomical assignment)	References
Rivers, Lake, pond, streams	16SrRNA	*unk	Phylum: Acidobacteria, Bacteroidetes, Chloroflexi, Firmicutes, Nitrospira, Proteobacteria, Verrucomicrobia Family: Burkholderiaceae, Erythrobacteraceae, Nitrospiraceae, Nitrosomonadaceae (SILVA database- version not available)	Hoellein et al. (2014)
Channel	16SrRNA	*unk	Phylum: Proteobacteria Order: Actinomycetales Family: Aeromonadacea, Campylobacteraceae, Flavobacteriaceae, Pseudomonadaceae, Rhodocyclaceae, Veillonellaceae Genus: *Arcobacter*, *Aeromonas*, *Pseudomonas* (SILVA database- version not available)	McCormick et al. (2014)
Streams	16SrRNA	PE, PP, PS	Phylum: Actinobacteria, Bacteriodetes, Chloroflexi, Firmicutes, Nitrospira, Planctomycetes, Proteobacteria Class: Betaproteobacteria, Gammaproteobacteria Family: Burkholderiaceae, Campylobacteraceae, Pseudomonadaceae, Veillonellaceae Genus: *Acinetobacter*, *Aquabacterium*, *Arcobacter*, *Azospira*, *Pseudomonas*, *Rheinheimera* (SILVA database- v119)	McCormick et al. (2016)
River	16SrRNA	PE, PP, PS	Phylum: Acidobacteria, Actinomycetales, Bacteroidetes, Sphingobacteriales Order: Myxococcale Family: Veillonellaceae, Aeromonadaceae, Campylobacteraceae, Chitinophagaceae, Hydrogenophilaceae, Methylococcaceae, Moraxellaceae, Pseudomonadaceae (SILVA database- version not available)	Hoellein et al. (2017)
River, WWTP	18SrRNA	HDPE, PS	Phylum: Ascomycota, Basidiomycota, Chytridiomycota, Cryptomycota, LKM15 Genus: *Candida*, *Chytridium*, *Cryptococcus*, *Kazachstania*, *Saccharomyces*, *Trichosporon* (SILVA database- v123)	Kettner et al. (2017)
River	16SrRNA	HDPE, PS	Family: Flavobacteriaceae, Erythrobacteraceae, Hyphomonadaceae, Methylophilaceae, Planctomycetaceae, Rhodobacteraceae, Verrucomicrobiaceae Genus: *Blastopirellula*, *Erythrobacter*, *Flavobacterium*, *Hyphomona*, *Methylotenera*, *Pirellula*, *Planctomyces*, *Sphingopyxis*, *Tenacibaculum* (SILVA database- v123)	Oberbeckmann et al. (2018)
Lake	16SrRNA	PE, PP	Phylum: Acidobacteria, Actinobacteria, Bacteroidetes, Chloroflexi, Cyanobacteria, Proteobacteria Class: Bacilli, Betaproteobacteria, Deltaproteobacteria, Gammaproteobacteria, Flavobacteriia Family: Aaerolineae, Oscillatoriophycideae, Synechococcophycideae (SILVA database- version not available)	Miao et al. (2019)
River	16SrRNA	PVC	Phylum: Actinobacteria, Acidobacteria, Bacteroidetes, Chlamydiae, Chlorobi, Fibrobacteres, Firmicutes, Gemmatimonadetes, Hydrogenedentes, Planctomycetes, Proteobacteria (SILVA database- version not available)	Wu et al. (2019)
Lake	16SrRNA	EPS, PA, PE, PP	Class: Alphaproteobacteria, Gammaproteobacteria Family: Sphingomonadaceae, Rhodobacteraceae, Burkholderiaceae Genus: *Acidovorax*, *Altererythrobacter*, *Aquabacterium*, *Hydrogenophaga*, *Ideonella*, *Leptothrix*, *Massilia*, *Novosphingobium*, *Porphyrobacter*, *Pseudorhodobacter*, *Rhodobacter*, *Sphingomonas*, *Sphingorhabdus* (SILVA database- v132)	Di Pippo et al. (2020)
River	16SrRNA	PBT, PE, PP, PS	Phylum: Actinobacteria, Bacterioidetes, Chloroflexi, Cloacimonetes, Cyanobacteria, Firmicutes, Proteobacteria Class: Bacteroidia, Gammaproteobacteria, Order: Rhodocyclales, Vibrionaceae Family: Alteromonadaceae, Nitrospirae, Nocardiaceae Genus: *Alilihoeflea*, *Acinetobacter* (Database not provided)	Xue et al. (2020)
River	16SrRNA	PE, PP	Phylum: Acidobacteria, Actinobacteria, Bacterioidetes, Chloroflexi, Cyanobacteria, Deinococcus-Thermus, Firmicutes, Planctomycetes, Proteobacteria, Verrucomicrobia (SILVA database- v132)	Wang et al. (2020)
Pond	16SrRNA	*unk	Phylum: Actinobacteria, Bacteroidetes, Cyanobacteria, Dependentiae, Proteobacteria, Verrucomicrobia Family: Acetobacteraceae, Burkholderiaceae, Caldilineaceae, Chthoniobacteraceae, Microcystaceae, Microscillaceae, Pseudanabaenaceae, Rhizobiaceae, Sphingomonadaceae, Xanthobacteraceae Genus: *Aquabacterium*, *Allorhizobium*, *Bradyrhizobium*, *Herbaspirillum*, *Neorhizobium*, *Pararhizobium*, *Rhizobium* (SILVA database- v132)	Wen et al. (2020)
Lake	WGS	PCL, PP, PS, PVC	Bacteria Phylum: Actinobacteria, Bacteroidetes, Chloroflexi, Cyanobacteria, Planctomycetes, Proteobacteria, Thaumarchaeota Class: Alphaproteobacteria, Gammaproteobacteria, Deltaproteobacteria, Flavobacteria Order: Alteromonadales, Desulfobacterales, Flavobacteriales, Rhodobacterales Family: Desulfobacteraceae, Flavobacteriaceae, Hyphomonadaceae, Pseudoalteromonadaceae, Rhodobacteraceae,Vibrionaceae Species: *Actibacterium atlanticum*, *Desulfatibacillum aliphaticivorans*, *Desulfatibacillum alkenivorans*, *Hyphomonas adhaerens*, *Hyphomonas jannaschiana*, *Muricauda* sp., *Nautella italica*, *Pseudooceanicola batsensis*, *Pseudoalteromonas shioyasakiensis*, *Thalassobius mediterraneus*, *Vibrio alginolyticus*, *Vibrio campbellii* Eukarya Families: Geminigeracea, Noelaerhabdaceae, Genus: *Emiliania*, *Thalassiosira* Archaea Genus: *Nitrosopumilus*, *Thaumarchaeota* (Database not provided)	Bhagwat et al. (2021)
WWTPs	16SrRNA	PA, PAN, PE, PET, PP, PS, silicones	Family: Comamonadaceae, Flavobacteriaceae, Rhodocyclaceae Genus: *Acidobacter*, *Aquaspirillum*, *Arenimonas*, *Byssovorax*, *Chryseobacterium*, *Dokdonella*, *Legionella*, *Ferruginibacter*, *Nannocystis*, *Nitrosomonas*, *Piscinibacter*, *Steroidobacter*, *Tolumonas*, *Terrimonas* (SILVA database- version not available)	Galafassi et al. (2021)
Lake	16SrRNA, 18SrRNA, ITS	HDPE, LDPE, PHB	Bacteria Phylum: Actinobacteria, Bacteroidetes, Cyanobacteria, Proteobacteria, Verrucomicrobia Family: Comamonadaceae, Moraxellaceae, Sphingomonadaceae Genus: *Mycoplana*, *Erythromicrobium*, *Rhodobacter*, *Rhodoferax*, *Zymomonas*, *Erythromicrobium*, *Pseudanabaena*, *Sphingomonas*, *Polaromonas* Eukarya Phylum:Ascomycota, Basidiomycota, Chytridiomycota, Ciliophora, Ochrophyta, Chlorophyta, Cryptophyta, Dinophyta Class: Chrysophyceae, Chytridiomycetes, Dinophiceae Genus: *Arrhenia*, *Betamyces*, *Chlamydomonas*, *Cryptococcus*, *Cryptomonas*, *Epipyxis*, *Malassezia*, *Paranamyces*, *Stentor*, *Tetraselmis*, *Uroleptus*, *Vorticella*, *Xylodon* (PR2 v.4.12 for 18S rRNA; Greengenes v.13.8 for 16S rRNA and UNITE v. 04.02.2020 for ITS)	González-Pleiter et al. (2021)
WWTPs	16SrRNA	PE, PP, PS	Family: Aeromonadaceae, Bacteroidaceae, Campylobacteraceae, Enterobacteriaceae, Lachnospiraceae, Moraxellaceae, Sphingomonadaceae Genus: *Acinetobacter*, *Arcobacter*, *Klebsiella*, *Sphingomonas* (SILVA database- version not available)	Kelly et al. (2021)
River	16SrRNA ITS	*unk	Bacteria Phylum: Acidobacteria, Actinobacteria, Bacteroidetes, Chlamydiae, Firmicutes, Planctomycetes, Proteobacteria, Verrucomicrobia Order: Acidimicrobiales, Actinomycetales, Bacillales, Chlamydiales, Desulfarculales, Gallionellales, Kiloniellales, Legionellales, Methylococcales, Methylophilales, Nitriliruptorales, Opitutales, Puniceicoccales, Rhodospirillales, Sphingomonadales, Tepidisphaerales Fungi Phylum: Ascomycota, Basidiomycota, Cercozoa, Chytridiomycota, Mortierellomycota, Rozellomycota (RDP classifier for 16S rRNA; UNITE database- version not available for ITS)	Li et al. (2021)
WWTPs	16SrRNA	LDPE, PCL, PHB, PET, PLA, POM, PS	Phylum: Actinobacteria, Bacteroidetes, Chloroflexi, Firmicutes, Planctomycetes, Proteobacteria, Saccharibacteria Family: Acidimicrobia, Alphaproteobacteria, Betaproteobacteria, Clostridia, Comamonadaceae, Gammaproteobacteria, Hyphomicrobiaceae, Moraxellaceae, Rhodobacteraceae, Rhodocyclaceae, Sphingobacteria Genus: *Acidovorax*, *Acinetobacter*, *Aquabacterium*, *Dodonella*, *Iamia*, *Leeia*, *Microthrix*, *Mycobacterium*, *Perludibaca*, *Pseudomonas*, *Roseiflexus*, *Sphaerotilus*, *Terrimonas*, *Variovorax*, *Zoogloea* (SILVA database- v128)	Martínez-Campos et al. (2021)
River	16SrRNA	PA, PE, PET, PP, PS, PVC, PU	Phyum: Bacteroidetes, Cyanobacteria, Proteobacteria Class: Betaproteobacteria, Chloroplast, Gammaproteobacteria Order: Burkholderiales, Flavobacteriales, Pseudomonadales Family: Comamonadaceae, Moraxellaceae, Sporichthyaceae Genus: *Acidovorax*, *Acinetobacter*, *Alkanindiges*, *Flavobacterium*, *Fluviicola*, hgcI clade, *Hydrogenophaga*, *Limnohabitans*, *Massilia*, *Pseudarcicella*, *Pseudomonas*, *Roseateles*, *Sediminbacterium*, *Simplicispira*, *Synechococcus*, *Thiothrix* (Database not provided)	Mughini-Gras et al. (2021)
Reservoir	16SrRNA	HDPE, PHBV, PLA	Phylum:Acidobacteria, Actinobacteria, Bacteroidetes, Cyanobacteria, Planctomycetes, Proteobacteria, Verrucomicrobia Class: Alphaproteobacteria, Betaproteobacteria, Gammaproteobacteria Family: Comamonadaceae Genus: *Azospirillum*, *Caldimonas*, *Caulobacter*, *Ideonella*, *Rhodobacter*, *Segetibacter*, *Tibeticola*, *Variovorax* (SILVA database- v138)	Nguyen et al. (2021)
River	16SrRNA	PET, PS, HDPE	Phylum: Acidobacteria, Actinobacteria, Flavobacteria, Nitrospira, Proteobacteria, Class: Acidimicrobiia, Actinobacteria, Alphaproteobacteria, Deltaproteobacteria, Gammaproteobacteria, Flavobacteria Order: Methylophilales, Rhizobiales Genus: *Planktophila* Species: *Limnobacter thiooxidans* (Database not provided)	Qiang et al. (2021)
River	ITS	PBT, PE, PP, PS	Phylum: Ascomycota, Basidiomycota, Blastocladiomycota, Chytridiomycota, Mucoromycota, Zoopagomycota Class: Dothideomycetes, Eurotiomycetes Genus: *Alternaria*, *Cladosporium*, *Eurotium*, *Lewia*, *Neocamarosporium*, *Paradendryphiella*, *Paraphoma*, *Phaeosphaeria*, *Phoma*, *Plectosphaerella*, *Rhodotorula*, *Vishniacozyma* (UNITE database-v7)	Xue et al. (2021)
River	16SrRNA ITS	PE, PP	Bacteria Phylum: Acidobacteria, Actinobacteria, Bacteroidetes, Cyanobacteria, Proteobacteria, Thermoleophilia, Verrucomicrobiae Class: Acidimicrobiales, Alphaproteobacteria, Anaerolineae, Betaproteobacteria, Gammaproteobacteria Planctomycetia, Phycisphaerae, Thermomicrobia Fungi Phylum: Ascomycota, Basidiomycota, Blastocladiomycota, Mucoromycota Class: Blastocladiomycetes, Dothideomycetes, Sordariomycetes (SILVA database- v128)	Wang et al. (2021)
Stream	16SrRNA 18SrRNA	PE, PP, PS, PVC	Bacteria Order: Armatimonadales, Burkholderiales, Candidatus Kaiserbacteria, Candidatus Nomurabacteria, Chitinophagales, Cytophagales, Flavobacteriales, Nitrospirales, Oligoflexales, Pirellulales, Planctomycetales, Rhizobiales, Rhodobacterales, Sphingomonadales, Steroidobacterales, Verrucomicrobiales Family: Chitinophagaceae, Comamonadaceae Species: *Acinetobacter lwoffii*, *Aeromonas hydrophila*, *Afipia broomeae*, *Enterobacter ludwigii*, *Klebsiella pneumoniae*, *Nocardia farcinica*, *Pseudomonas aeruginosa* Eukarya Phylum: Bacillariophyta, Bicosoecida, Chlorophyta, Ciliophora, Cryptomycota, Gastrotricha, Holozoa, Nematodes, Peronosporomycetes, Rotifera Family: Bacillariophyceae, Haptoria, Heterotrichea, Hypotrichia, Oligohymenophorea, Phyllopharyngea, Prostomatea Species: *Chaetophora incrassata*, *Microspora* sp., *Oedocladium prescottii*, *Radiococcus* sp. (SILVA database- v138)	Weig et al. (2021)
River	16SrRNA	PE, PS	Phylum: Bacteroidetes, Betaproteobacteria, Cyanobacteria, Deinococcus-Thermus Genus: *Acinetobacter*, *Chamaesiphon*, *Clostridium*, *Deinococcus*, *Ensifer*, *Hymenobacter*, *Novispirillum*, *Paenibacillus* (Greengenes database-version not available)	Delacuvellerie et al. (2022)
Lake	18SrRNA	PE, EPS, PP	Phylum: Charophyta, Chlorophyta, Ciliophora, Stramenopiles Class: Copepoda, Monogononta, Ploimida Order: Adinetida, Chaetonotida, Pennales, Peronosporomycetes Family: Bacillariophyceae, Desmidiaceae, Scenedesmaceae, Ulvellaceae Genus: Ceratium, Gonyaulex, Peridinium Species: *Legionella* spp., *Pseudomonas aeruginosa*, *Salmonella* spp. (SILVA database- v132)	Di Pippo et al. (2022)
River	16SrRNA 23SrRNA ITS	EPS, LDPE, PVC	Bacteria Phylum: Actinobacteria Class: Alphaproteobacteria, Bacteroidetes, Betaproteobacteria, Gammaproteobacteria Family: Enterobacteriaceae Genus: *Aeribacillus*, *Halomonas* Eukarya Phylum: Ascomycota, Basidiomycota, Stramenopiles (SILVA database for 16SrRNA and 23S rRNA- version not available; UNITE for ITS- version not available)	Chaudhary et al. (2022)
Pond	16SrRNA WGS	PA	Phylum: Actinobacteriota, Bacteroidota, Cyanobacteria, Firmicutes, Proteobacteria Genus: *Nitrososphaera*, *Nitrosospira*/*Nitrosomonas*/*Nitrosococcus*, *Nitrobacter*, *Nitrospira*, *Thiobacillus* Species: *Candidatus Nitrospira inopinata*, *Dechloromonas denitrificans*, *Nitrobacter hamburgensis*, *Nitrosomonas europaea*, *Nitrososphaeraceae archaeon*, *Nitrospira moscoviensis*, *Thiobacillus denitrificans* (Database not provided)	Huang et al. (2022)
Rivers	16SrRNA	PE, PS	Phylum: Bacteroidetes, Cyanobacteria, Firmicutes, Proteobacteria Class: Bacilli, Bacteroidia, Betaproteobacteria, Clostridia, Deltaproteobacteria, Flavobacteriia, Gammaproteobacteria Species: *Limnothrix redekei*, *Arcobacter cryaerophilus*, *Bacillus cereus*, *Brevundimonas naejangsanensis*, *Comamonas testosterone*, *Diaphorobacter oryzae*, *Glutamicibacter protophormiae*, *Parabacteroides chartae* (SILVA database- v138)	Nguyen et al. (2022)
Lake, river	16SrRNA 18SrRNA	PET, SBP	Bacteria Phylum: Acidobacteria, Chloroflexi, Cyanobacteria, Firmicutes, Proteobacteria, Verrucomicrobia Eukarya Phylum: Ciliophora (Database not provided)	Li W. et al. (2023)
River	16SrRNA 18SrRNA	LDPE, PET, PS, PVC	Bacteria Phylum: Bacteroidetes, Cyanobacteria, Proteobacteria Class: Alphaproteobacteria, Bacteroidia, Gammaproteobacteria, Oxyphotobacteria Family: Burkholderiaceae, Chitinophagaceae, Hyphomicrobiaceae, Methylomonaceae, Methylophilaceae, Microtrichaceae, Pirellulaceae, Rhodobacteraceae, Rhodocyclaceae, Saprospiraceae, Sphingomonadaceae Eukarya Phylum: Annelida, Bryozoa, Chloroplastida, Mollusca, Ochrophyta, Platyhelminthes, Stramenopiles Class: Clitellata, Gastropoda, Phylactolaemata, Rhabditophora Family: Aspidiscidae, Caecidae, Chaetophoraceae, Cocconeidaceae, Cyprididae, Erpobdellidae, Gomphonemataceae, Monostromataceae, Naididae, Opisthonectidae, Planariidae, Scopalinidae, Stenostomidae (SILVA database- v128)	Martínez-Campos et al. (2023)
Lake	16SrRNA 18SrRNA	PE, PLA + PBAT, PP	Bacteria Phylum: Acidobacteria, Actinobacteria, Bacteroidetes, Chlamydiae, Chloroflexi, Cyanobacteria, Firmicutes, Gemmatimonadetes, Nitrospirae, Omnitrophicaeota, Spirochaetes, Planctomycetes, Proteobacteria, Verrucomicrobia Eukarya Subphylum: Ochrophyta (Database not provided) Archaea Phylum: Euryarchaeota	Miao et al. (2023)
Reservoir	16SrRNA	HDPE, PHBV, PLA	Family: Acetobacteraceae, Bacteriovoracaceae, Caulobacteraceae, Cellvibrionaceae, Chitinophagaceae, Comamonadaceae, Crocinitomicaceae, Elsteraceae, Flavobacteriaceae, Gemmatimonadaceae, Oxalobacteraceae, Polyangiaceae, Pedosphaeraceae, Rhodobacteraceae, Saprospiraceae, Spirosomaceae Genus: *Aetherobacter*, *Asticcacaulis*, *Caulobacter*, *Cellvibrio*, *Chitinimonas*, *Elstera*, *Emticicia*, *Flavobacterium*, *Ferruginibacter*, *Fluviicola*, *Gemmatimona*, *Parasediminibacterium*, *Pajaroellobacter*, *Pedosphaera*, *Peredibacter*, *Pseudomonas*, *Rhodoferax*, *Rhodovastum*, *Undibacteria* (SILVA database- v138)	Nguyen et al. (2023)
River	16SrRNA 23SrRNA	PET, PLA	Bacteria Phylum: Proteobacteria, Actinobacteria, Bacteroidota, Chloroflexi, Cyanobacteria, Firmicutes, Ignavibacteriae, Verrucomicrobia Eukarya Phylum: Bacillariophyta, Chlorophyta, Euglenozoa (Database not provided)	Song et al. (2023)
River	16SrRNA	HDPE, PP, PVC	Phylum: Actinobacteriota, Bacteroidetes, Cyanobacteria, Firmicutes, Proteobacteria Genus: *Aeromonas*, *Bacillus*, *Chloroplast*, *Enterobacter*, *Escherichia*, *Hydrogenophagat*, *Listeria*, *Lutolibacter*, *Pseudorhodobacter*, *Rhodoferax*, *Shigella*, *Sphaerotilus*, *Tychonema* Species: *Citrobacter freundii*, *Campylobacter*, *Enterobacter* spp., *E. coli*, *Klebsiella pneumoniae*, *L. monocytogenes*, *Mammaliicoccus vitulinus*, *Providencia rettgeri* (SILVA database- v138)	Witsø et al. (2023)
River	16SrRNA	PE, PET	Class: Acidimicrobiia, Acidobacteriia, Alphaproteobacteria, Bacilli, Bacteroidia, Bdellovibrionia, Cyanobacteriia, Chloroflexia, Gammaproteobacteria, Gemmatimonadetes, NB1-j, Oligoflexia, Vicinamibacteria (SILVA database- v138)	Xu et al. (2023)
River	16SrRNA	PE, PLA, PS, PVC	Phylum: Bacteroidetes, Firmicutes, Nitrospirae, Proteobacteria, Verrucomicrobia Class: Alphaproteobacteria, Betaproteobacteria, Gammaproteobacteria, Deltaproteobacteria, Nitrospira Family: Comamonadaceae, Methylophilaceae, Rhodocyclaceae Genus: *Methylotenera*, *Methyloversatilis*, *Nevskia*, *Rubrivivax* (Database not provided)	Zhu et al. (2023)
Lake	WGS	nylon, PET, PMMA, PVA, PVAC	Bacteria Class: Alphaproteobacteria, Bacteroidia, Gammaproteobacteria, Verrucomicrobiae Genus: *Bradyrhizobium*, *Hydrogenophaga*, *Mesorhizobium*, *Phyllobacterium*, *Pseudolabrys*, *Sediminibacterium*, *Variovorax* Species: *Burkholderia cenocepacia*, *Pseudomonas aeruginosa*, *Pseudomonas syringae*, *Salmonella enterica*, *Xanthomonas oryzae* Eukarya Family: Hominidae, Plasmodiidae, Sarcocystidae Archaea Family: Haloarculaceae, Halobacteriaceae, Halorubraceae, Natrialbaceae (Kraken database)	Xu et al. (2024)
Lake	16SrRNA 18SrRNA	PBAT, PBS, PE, PHA, PLA, PP, PS, PVC	Bacteria Class: Alphaproteobacteria, Anaerolineae, Gammaproteobacteria, Nitrospiria, Polyangia, Pseudomonadales, Rhizobiales, Vicinamibacteria Eukarya Class: Clitellata (Database not provided)	Zhang et al. (2024)

*unk: unknown. **WGS: whole genome sequencing.

Most of the available information on freshwater plastisphere biodiversity comes from culture-independent methods (Table 1), which allow a comprehensive characterization of plastisphere-associated microbiomes. Among them, the use of high-throughput sequencing methods, both as amplicon sequencing of SSU RNA genes and shotgun metagenomic sequencing, are essential to decipher the taxonomic and functional diversity of samples, thus providing the composition and the metabolic potential of the entire microbial community (Dey et al., 2022; Wani et al., 2023). Recent developments in sequencing techniques have led to sequence very long reads (up to 50 kb) offering multiple cutting-edge options for understanding microbiome structure and functioning (Tedersoo et al., 2021). For example, when applied to amplicon sequencing (e.g., 16S rRNA gene), long-reads can resolve microbial taxonomy at deeper levels rather than short-reads due to the ability to read the entire gene with single nucleotide resolution leading to the identification of sub-species clades or “strains” within the community. Most studies have focused on Bacteria, with very few reports on archaeal and eukaryotic biodiversity (Table 1).

Proteobacteria, Bacteroidetes, Actinobacteria, Firmicutes, Verrucomicrobia, Planctomycetes, and Acidobacteria were the bacterial phyla most frequently detected on plastic particles in freshwaters (Table 1). Plastisphere bacteria are mainly affiliated with Gammaproteobacteria (family Burkolderaceae), Alphaproteobacteria (e.g., families Sphingomonadaceae, Rhodobacteraceae, and Hyphomicrobiaceae), Flavobacteria, and Firmicutes (Table 1). Rhodobacteraceae and Burkolderaceae are recurrent, particularly owing to their role as initial colonizers (Polz et al., 2006; Bhagwat et al., 2021; Di Pippo et al., 2022). Their involvement in different biogeochemical cycles and their mutualisms with eukaryotes suggest an important role in microbial community succession on plastic surfaces (Simon et al., 2017).

Archaea are likely to represent a minor component of the plastic-associated microbial community (<0.1% of total amplicon sequences) (Mughini-Gras et al., 2021), showing a lower diversity compared to Bacteria.

Microscopy observations indicated that freshwater eukaryotic microbes of various trophic levels may represent a significant portion of plastisphere biodiversity (Carson et al., 2013; Oberbeckmann et al., 2014; Bryant et al., 2016; Masó et al., 2016), although high-throughput sequencing data are still limited (Kettner et al., 2017; Bhagwat et al., 2021; González-Pleiter et al., 2021; Li et al., 2021; Wang et al., 2021; Weig et al., 2021; Xue et al., 2021; Chaudhary et al., 2022; Di Pippo et al., 2022; Li W. et al., 2023; Martínez-Campos et al., 2023; Miao et al., 2023; Song et al., 2023; Xu et al., 2024; Zhang et al., 2024). Different taxa of primary producers (Chlorophyta, Charophyta, Bacillariophyta), primary/secondary consumers (Peritrichia, Oligotrichia), mixotrophs (Dinophyceaea), saprotrophic/parasitic fungi (Cryptomycota, Peronosperales, Oomycetes), and metazoan consumers were retrieved (Table 1). In recent studies various conventional and bio-based plastic polymers (e.g., postconsumer plastic, “raw” plastic from known manufacturing sources) were used to assess microbial plastic colonization in freshwaters under different field and laboratory settings (Table 1). Regardless of the varying tested conditions, different environmental, spatial, and temporal factors (e.g., redox potential, salinity, nutrient concentration, geographical location, anthropogenic influence, seasonality) appeared to drive the microbial composition and assembly of the freshwater plastisphere (Zhang et al., 2004; Kettner et al., 2017; Bhagwat et al., 2021; González-Pleiter et al., 2021; Li et al., 2021; Weig et al., 2021; Xue et al., 2021; Di Pippo et al., 2022; Martínez-Campos et al., 2023; Miao et al., 2023; Xu et al., 2024). The role of polymer types and properties is unclear and currently under debate (Jacquin et al., 2019; Bhagwat et al., 2021; Coons et al., 2021; Delacuvellerie et al., 2021; Mughini-Gras et al., 2021; Weig et al., 2021; Sooriyakumar et al., 2022; Wang et al., 2022; Li K. et al., 2023; Miao et al., 2023), since only few studies have reported that specific plastic polymers can select different communities (McCormick et al., 2016; Di Pippo et al., 2020; Delacuvellerie et al., 2021; Li et al., 2021; Martínez-Campos et al., 2021; Mughini-Gras et al., 2021). Surface properties such as roughness, topography, and electrostatic charge are known to influence freshwater bacterial attachment and biofilm assembly (Rummel et al., 2017; Nguyen et al., 2021). Microbial colonization during the early developmental stages and the subsequent microbial biofilm maturation are directly influenced by the chemical, physical, mechanical, and morphological properties of the polymer substrata (Dexter, 1979; Rummel et al., 2017; Kreve and Reis, 2021; Nguyen et al., 2021; Zheng et al., 2021; Jia, 2022). On the one hand, the presence of plastic additives (e.g., plasticizers, flame retardants, colorants) can promote hydrophobicity and alter the original properties of the bare solid surfaces (Karlsson et al., 1988; De Tender et al., 2015; Bhagwat et al., 2021). On the other hand, environmental aging, weathering, and photo-oxidation can reduce the surface hydrophobicity, thus promoting microbial colonization (Gong et al., 2019; Bao et al., 2022). The surface colonization processes involve a succession of microorganisms that contribute to the establishment of a stable biofilm consortium. By producing extracellular polymeric substances (EPS), pioneer microorganisms can facilitate their attachment to surfaces, but also provide a suitable carbon source for other microbial species (Yang Y. et al., 2020). Consequently, early colonizers can be outcompeted by other taxa with increasing duration of exposure to environmental conditions (e.g., incubation time in water), thus leading to converging community composition over time on different materials (Pinto et al., 2019; Yang Y. et al., 2020; Nguyen et al., 2021; Chaudhary et al., 2022; Martínez-Campos et al., 2023; Miao et al., 2023; Xu et al., 2023).

## 3 Ecosystem and human health-related issues: occurrence of plastic-associated pathogens and genetic elements of health concern

**Figure 1:**
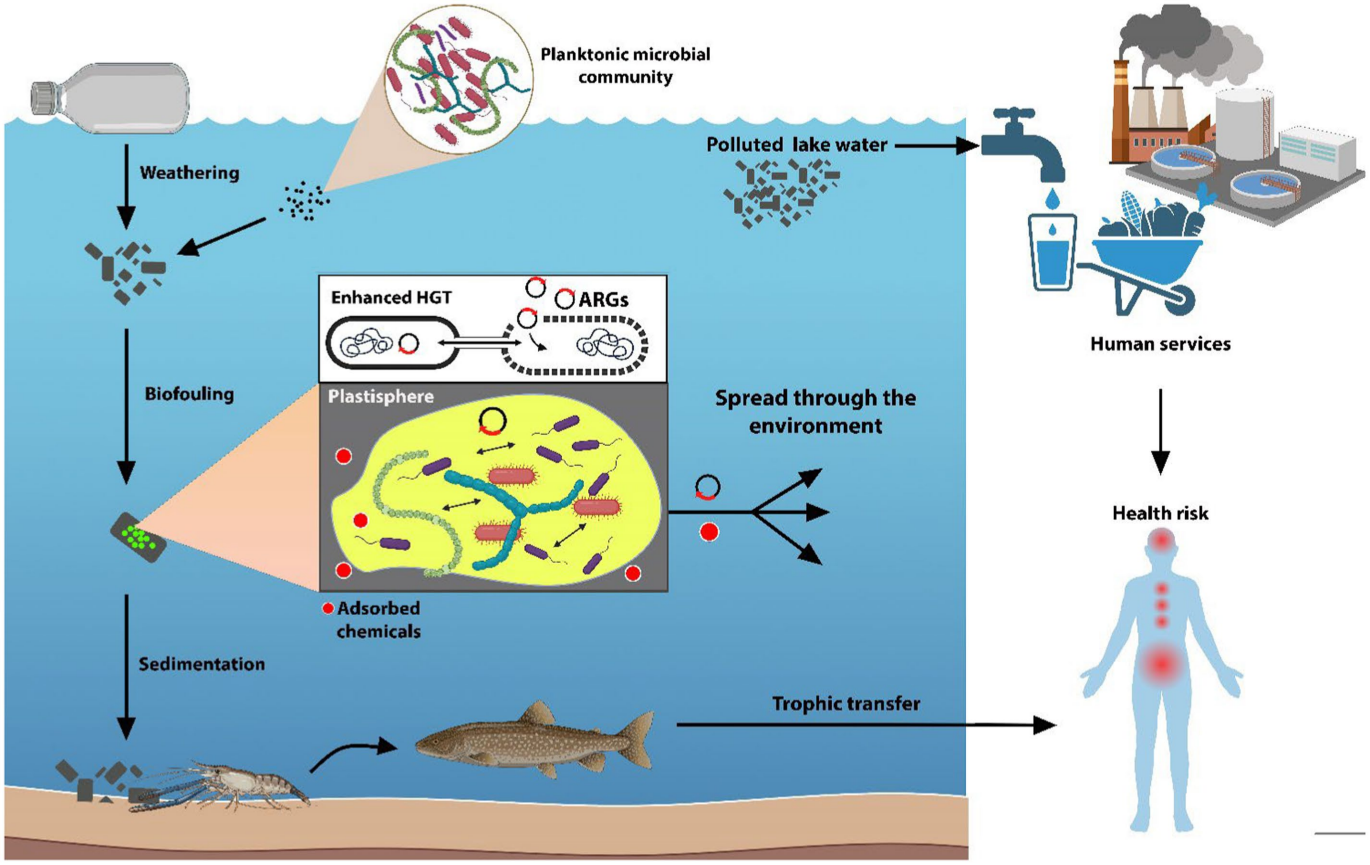
Pathways through which plastisphere and plastics may affect ecosystems and human health. Plastics released in freshwater as vectors for both microorganisms and chemicals adsorbed on their surfaces. Once in water, plastics are affected by abiotic factors leading to their fragmentation into smaller particles, that are colonized by the planktonic microbial community (biofouling) with an increase of density of the particles, causing their sedimentation through the water column. The close relationship between the biofilm microorganisms lead to an enhanced horizontal gene transfer (HGT) with the spreading of the ARGs through the plastisphere members. As ubiquitous pollutants, plastic debris travel throughout the environment leading to the spread of antibiotic resistance and adsorbed chemicals into freshwater, finally reaching services provided to human health and society. The persistent plastic debris can also enter the trophic chain. (Images created using BioRender.com.)

Plastic-associated microbiological elements of health concern are rarely monitored in freshwater ecosystems, despite their fundamental services provided to human health and society (e.g., drinking water supply, agricultural/industrial activities, recreational activities). Plastic debris and associated biofilms have been reported to represent newly available ecological niches that facilitate the accumulation of various harmful microbes. Recent studies have highlighted the presence of potentially pathogenic bacteria in freshwater plastisphere communities, including members of the genera *Vibrio*, *Pseudomonas*, *Acinetobacter*, *Arcobacter*, *Bacillus*, *Aquabacterium*, *Mycobacterium*, *Aeromonas*, *Tenacibaculum*, *Escherichia*, *Klebsiella*, and *Legionella* (Kirstein et al., 2016; McCormick et al., 2016). These bacteria can pose a significant risk to aquatic life and human health by causing infections, skin irritation, and even systemic diseases. In addition to bacterial pathogens, the plastisphere can also harbor eukaryotic microorganisms that can have a potential negative impact. Potentially toxic microalgae and potentially pathogenic fungi (i.e., Chytridiomycota and Cryptomycota species) were found on plastic debris, raising concerns about its role in promoting harmful algal blooms and the spread of water-borne fungal diseases (Barros and Seena, 2021; Di Pippo et al., 2022). More recently, several studies on plastic and MP dispersal in freshwaters showed the co-presence of pathogens and Mobile Genetic Elements (MGEs), including ARGs, thus suggesting a higher probability of antibiotic resistance acquisition mediated by the plastisphere (Junaid et al., 2022; Table 2). Considering the worldwide spread of MPs in the environment, ARGs presence on MPs may exacerbate risk for human to acquire ARGs and-or resistant microorganisms of health concern. Indeed, some studies have revealed that marine microorganisms can uptake MPs from the water environment transferring in the food chain and more recently has been shown that ARGs can transfer through the trophic level into the food chain (Zhu et al., 2019; Figure 1).

**Table 2:** ARGs and ARBs detected in plastic-associated biofilms in freshwater ecosystems.

Freshwater source	Plastics	Target genes/pathogens	Main results	Techniques	References
Lake, WWTP	PS	ARGs	ARGs: *intI1*	qPCR	Eckert et al. (2018)
River	PVC	ARGs and Pathogens	ARGs: multidrug-ARGs, MLS, bacitracin, polymyxin, acriflavine, beta-lactam, aminoglycoside. Pathogens: *Pluralibacter*, *Pseudomonas*, *Leclercia*, *Pantoea*.	Metagenomic and metatranscriptomic sequencing	Wu et al. (2019)
River	PE, PP	ARGs	ARGs: aminoglycoside residence genes (*aadA1* and *strB*), macrolide residence genes (*mefA*, *ermB*, *ermC* and *ermE*), chloramphenicol residence genes (*cfr, cmlA, fexA, fexB and floR*), sulfonamide residence genes (*sul1*, *sul2*, and *sul3*), and tetracycline residence genes (*tetA*, *tetBP*, *tetG*, *tetH*, *tetM*, *tetO*, *tetQ*, *tetS*, *tetT*, *tetW*, *tetX*), integrase genes (*intI1* and *intI2*)	qPCR	Wang et al. (2020)
Urban water	HDPE	ARGs and Pathogens	ARGs: aminoglycosides, β-lactams, fluoroquinolones, multidrugs, macrolide-lincosamide-streptogramin B (MLSB), sulfonamides, tetracycline, trimethoprim, and vancomycin resistance genes. Pathogens: *Mycobacterium abscessus*, *Bacillus megaterium*, *Mycobacterium gilvum*, *Listeria monocytogenes*, *Pseudomonas putida*, *Pseudomonas mendocina*.	HT-qPCR	Yang K. et al. (2020)
Lake	HDPE, LDPE, PHB	ARGs	ARGs: *sul1*, *ermB*	qPCR	González-Pleiter et al. (2021)
River	PB, PE, PP	ARGs, MGE, HPB	ARGs and MGE: sulfonamides (sul1, sul2), tetracyclines (tetA, tetB, tetM, tetW), quinolones (qnrB and qnrS), macrolides (ermB and ermF) resistance genes and mobile genetic element (MGE, intI1). HPB: *Streptococcus mitis*, *Pseudomonas fluorescens*, *Pseudomonas savastanoi*, *Klebsiella pneumoniae*, *Pseudomonas putida*, Pseudomonas entomophila, Pseudomonas protegens, *Pseudomonas stutzeri*, *Salmonella enterica*, and *Aeromonas hydrophila*.	qPCR, 16S rRNA	Hu et al. (2021)
Lakes	EPS, PE, PP	ARGs and Pathogens	ARGs: *intI1* Pathogens: *Legionella* spp., *Pseudomonas aeruginosa*	qPCR, LAMP-PCR	Di Pippo et al. (2022)
River, reservoir, bay	PE, PS	ARGs, MGEs, Pathogens	ARGs: Aminoglycoside, Beta-Lactamase, Diaminopyrimidine, Multidrug, Sulfonamide, Tetracycline, Fluoroquinolone, MLSB, Vancomycin MGEs: Transposase, Plasmid, Integrase, Insertional Pathogens: *Mycobacterium* sp., *Mycobacterium smegmatis*, *Mycobacterium gilvum*, *Mycobacterium abscessus*, *Klebsiella pneumoniae*, *Enterobacter cloacae*	HT-qPCR, 16S rRNA	Li H. et al. (2022)
River	*unk	ARGs, VFs	ARGs: *macB* (MLS), *tetA* (tetracycline), *novA* (aminocoumarin), *bcrA* (peptide) VFs: *mgtC*, mu-toxin	Metagenomics	Li R. et al. (2022)
River	PLA, PVC	ARGs, Pathogens	ARGs: macrolides (macB), multidrug (ceoB), macrolide-lincosamide-streptogramin B (macB, mefA), chloramphenicol (floR), sulfonamide (sul1, sul2), tetracycline (tetA, tetG, tetM, tetO, tetQ, tetW), beta-lactam (blaOXA and blaTEM), fluoroquinolone (mfpA), bacitracin, rifampicin, acriflavine	Metagenomics	Wu et al. (2022)
River	PA, PE, PET, PMMA, PP	ARGs and MGEs	ARGs: Multidrug, Bacitracin, Sulfonamide, Tetracycline, Chloramphenicol, Rifamycin and Vancomycin resistance genes. MGEs: plasmid, transposase, insertion sequence transposase (IST), insertion sequence (IS), and integrase.	Metagenomics	Xu et al. (2022)
Lake, canal and river	PBAT, PET	ARGs	ARGs: tetracycline (*tetA*, *tetB*, *tetC*, *tetG*, *tetM*, *tetQ*, *tetX*), quinolone (*qnrA*, *qnrB*, *qnrS*), sulfonamide (*sul1*, *sul2*), lactam (blaOXA10, blaQ), macrolide (*ermB*, *mefA*), erythromycin (*ereB*), chloramphenicol (*cmlA1*), multidrug-resistant genes (NDM-1), and new multidrug-resistant genes (MCR-1). MGEs: *intI1*, *tnpA04*, *tnpA05*.	qPCR	Zhou et al. (2022)
River	LDPE, PET, PS, PVC	ARGs	ARGs: erythromycin (*ermF*), sulphonamide (*sul1*), trimethoprim (*dfrA1*), quinolone (*qnrSrtF11A*)	qPCR	Martínez-Campos et al. (2023)
River	PLA, PET	ARGs	ARGs: *qnrS, blaNDM-1*, *FloR*, *sul1*, *qnrA*, *tetG*, *mcr-1*	qPCR	Chen et al. (2024)

ARG enrichment in plastic-associated biofilms is promoted by the proximity and close contact between bacterial cells that facilitate horizontal gene transfer, contributing to long-distance dispersal and long-term persistence of ARGs in the environment (Di Pippo et al., 2022; Du et al., 2022; Rubin and Zucker, 2022; Luo et al., 2023; Chen et al., 2024; Li et al., 2024; Figure 1). Moreover, the presence of plastic-adsorbed xenobiotics and metals might enhance ARG occurrence through co-selection processes (Abe et al., 2020; Junaid et al., 2022; Michaelis and Grohmann, 2023).

Unlike in marine environments, studies in freshwater showed limited plastisphere enrichment of ARGs compared to the surrounding waters and natural substrata (Wu et al., 2019; Wang et al., 2020; Xu et al., 2022), with no differences in ARGs and MGEs observed between plastisphere and natural biofilms (Hu et al., 2021). Recent studies on freshwater plastisphere have mainly analyzed the differences in ARG abundance and diversity by comparing (i) surface waters and other natural surfaces (González-Pleiter et al., 2021; Xu et al., 2022; Martínez-Campos et al., 2023), (ii) biodegradable and non-biodegradable plastics (Zhou Q. et al., 2022), (iii) different stages of biofilm development at different contamination levels (Table 2). Although clearly showing the worldwide spread of plastic-associated pathogens and ARGs, the information available on freshwater plastisphere is still limited to properly evaluate human health risks (Manaia, 2017; Zhang et al., 2021).

Notably, the identification of potential pathogens is at the genus level, which does not provide direct evidence of the pathogen’s occurrence, infectivity or virulence (Liu et al., 2022). Furthermore, quantitative PCR-based methods are limited to known functional genes and may miss novel or uncharacterized ARGs (Li et al., 2015). Combined omics approaches can provide detailed information on the collection of ARGs within the entire microbial community, namely the resistome (Bengtsson-Palme et al., 2018). However, due to the large diversity of ARGs and their incomplete coverage by the applied monitoring methods, the plastic-associated ARGs profiles are hardly comparable between different studies.

Recent publications showed how long-reads sequencing can help to overcome these technical limitations (Zhang et al., 2022). Long-read sequencing techniques improve the quality and completeness of metagenome-assembled genomes allowing to reduce errors and improving the accuracy of ARG identification and characterization (Table 2).

## 4 The role of plastisphere in plastic biodegradation processes

Once plastic items are transported through the aquatic environment, abiotic factors can cause changes in their mechanical and physico-chemical properties (Luo et al., 2022) and plastisphere microorganisms can modify MP surface properties by degrading additives, secreting MP-modifying/degrading enzymes. The plastisphere-mediated biodegradation of plastic debris and MPs is a complex multifaceted process in which polymers are first bio-fragmented through the secretion of extracellular enzymes. In the subsequent assimilation phase, the small and water-soluble molecules produced during the depolymerization of plastics are transported through the cell membrane.

Once inside the cell, plastic-derived molecules can be used as a carbon source to produce biomass and energy before being mineralized to CO_2_/CH_4_ and H_2_O (Tiwari et al., 2020; Yuan et al., 2020; Zeenat et al., 2021; Priya et al., 2022; Zhou Y. et al., 2022; Sun et al., 2023).

Plastic-degrading enzymes and microorganisms have been identified using culture-based approaches in which selected strains, isolated from environmental samples, are grown and screened for plastic-degrading activity under laboratory conditions (Mierzwa-Hersztek et al., 2019; Mohanan et al., 2020; Tiwari et al., 2020, 2022; Yuan et al., 2020; Amobonye et al., 2021; Nguyen et al., 2021; Othman et al., 2021; Priya et al., 2022; Zhou Y. et al., 2022). Microorganisms exhibiting plastic-degrading activity are typically isolated and enriched in a plastic-containing medium, while polymer-degrading enzymes are conventionally identified using a combination of biochemical and biomolecular approaches (Viljakainen and Hug, 2021; Herbert et al., 2022; Tiwari et al., 2022; Zhu et al., 2022; Tournier et al., 2023). To date, various genes and enzymes have been found to be associated with the plastisphere, including PETase (Polyethylene terephthalatease), MHETase (Mono (2-hydroxyethyl) terephthalate hydrolase), cutinases, lipases, oxidoreductases, laccases, peroxidases, and esterases (Tournier et al., 2023). In addition, several genes and enzymes enabling the breakdown of aromatic compounds into simpler and less toxic forms have been found in the plastisphere, mostly including dioxygenases (Seo et al., 2009).

More recently, the focus has shifted from studying specific strains or enzymes to examining the plastisphere community as a whole “degradation unit” (Jacquin et al., 2019; Yuan et al., 2020; Eronen-Rasimus et al., 2022; Joshi et al., 2022; Taipale et al., 2022; Cai et al., 2023; Maheswaran et al., 2023; Miao et al., 2023; Niu et al., 2023; Vaksmaa et al., 2023; Yu et al., 2023; Zhu et al., 2023). Various key microbial taxa can cooperate and show enzymatic potential for polymer biodegradation (Li K. et al., 2023; Li W. et al., 2023; Miao et al., 2023), also promoting changes in surface material properties, including chemical composition, morphology roughness, formation of holes and cracks, and weight loss. Such microbial driven surface features were assessed by scanning electron microscopy (SEM), Fourier-transform infrared spectroscopy (FTIR), atomic force microscopy, contact angle analysis, calorimetry, and mechanical testing (Chen et al., 2020; Denaro et al., 2020; Kosiorowska et al., 2022; Li J. et al., 2023; Sun et al., 2023). Several studies have shown that the presence of taxa associated with the biodegradation process is strictly connected to environmental factors such as light exposure (including UV radiation), heat, humidity, absorbed chemicals, pH, and oxygen levels, depending on the specific sampling sites (Tiwari et al., 2020; Yuan et al., 2020; Martínez-Campos et al., 2021; Vincent et al., 2022; Li W. et al., 2023). However, although mediated by biofilm composition and development (Miao et al., 2021; for further details see Sun et al., 2023), plastic polymer type and properties as surface morphology, topography, hydrophobicity, electric charge distribution, molecular weight, mobility, crystallinity, types of functional groups, additives, and plasticizers were likely the main abiotic factors affecting the overall biodegradation process (Tiwari et al., 2020; Yuan et al., 2020; Song et al., 2023).

Few studies currently available report inconsistent results on the direct involvement of plastisphere in biodegradation processes. The metabolic potential to hydrolyse and use the plastic polymers as carbon sources was not convincingly demonstrated, while plastic materials were mostly used as adhesion surfaces by opportunistic aquatic microbes (Oberbeckmann et al., 2021; Di Pippo et al., 2023). Further investigations are thus needed to provide a deeper understanding of plastisphere role in plastic biodegradation.

Advanced culture-independent approaches based on sequencing technologies are accelerating discoveries in this field. Although still in their infancy, “plastic-omics” (Viljakainen and Hug, 2021) are emerging as important tools for understanding the functional potential of the plastisphere, providing important insights into the identification of potentially degrading bacterial taxa, the factors influencing their enrichment, and the plastic degrading genes/enzymes, and thus a holistic understanding of the plastic degradation process (Viljakainen and Hug, 2021; Malik et al., 2023). Metatranscriptomics can be a powerful approach to reveal the gene expression profiles and transcriptional activity of microorganisms associated with plastic surfaces, elucidating metabolic pathways and gene regulatory networks involved in plastic biodegradation (Gilbert et al., 2008; Kirstein et al., 2016; Xu et al., 2019; Yang et al., 2019; Lu et al., 2020).

## 5 Conclusion

This review paper sheds light on the intricate relationship between plastic pollution and microbial communities in freshwater ecosystems, specifically focusing on the freshwater plastisphere. While molecular methods have expanded our understanding of plastisphere biodiversity, fundamental questions regarding the influence of the polymer type and properties and environmental factors on plastisphere structure, biodiversity and on community assembly remain unanswered. The presence of potentially pathogenic microbes and genetic elements of concern within the plastisphere raises important implications for ecosystem and human health. However, the extent of these risks and their impacts are still not fully elucidated, necessitating further research efforts. Advanced sequencing technologies offer promising avenues for uncovering the functional potential of the plastisphere, including its role in plastic biodegradation processes. Overall, the findings underscore the urgent need for comprehensive investigations into freshwater plastisphere dynamics, which are crucial for informing effective management strategies to mitigate the environmental and health impacts of plastic pollution in freshwater ecosystems.

## Author contributions

VB: Writing – review & editing, Writing – original draft. SG: Conceptualization, Writing – review & editing, Writing – original draft. CL: Writing – review & editing, Writing – original draft. SC: Writing – review & editing. SA: Conceptualization, Writing – review & editing. RC: Writing – review & editing. BM: Writing – review & editing, Writing – original draft. SR: Conceptualization, Writing – review & editing. FP: Conceptualization, Writing – review & editing, Writing – original draft.

## Glossary

ARBs: Antibiotic-resistant bacteria
ARGs: Antibiotic-resistance genes
ATR: Attenuated total reflectance
CLSM: Confocal Laser Scanning Microscopy
COD: Chemical Oxygen Demand
DSC: Differential Scanning Calorimetry
EPS: Expanded PolyStrene
ESI: Electrospray Ionization
FE-SEM: Field-emission Scanning Electron Microscopy
FID: Flame-ionization detector
FT-IR: Fourier transform infrared spectroscopy
GC: Gas chromatography
GPC: Gel permeation chromatography
HDPE: High density polyethylene
HPB: Human pathogenic bacteria
HT-qPCR: High-throughput qPCR
HTS: High-throughput screening
LAMP-PCR: Loop-mediated isothermal amplification PCR
LC: Reversed phase liquid chromatography
LDPE: Low density polyethylene
MB: MaterBi
MGEs: Mobile genetic elements
MPs: Microplastics
MS/MS: Tandem mass spectrometry
PA: Polyamide
PAN: Polyacrylonitrile
PB: Polybutylene
PBAT: Polybutylene adipate-co-terephthalate
PBS: Polybutylene succinate
PBT: Polybutylene terephthalate
PCL: Polycaprolactone
PE: Polyethylene
PET: Polyethylene terephthalate
PHA: Polyhydroxyalkanoates
PHB: Poly-3-hydroxybutyrate
PHBV: Poly (3-hydroxybutyrate-co-3hydroxyvalerate)
PLA: Polylactic acid
PMMA: Polymethyl methacrylate
POM: Polyoxymethylene
PP: Polypropylene
PS: Polystyrene
PU: Polyurethane
PVA: Polyvinyl alcohol
PVAC: Polyvinyl acetate
PVC: Polyvinylchloride
qPCR: Quantitative polymerase chain reaction
SBP: Starch-based plastics
SEM: Scanning electron microscopy
TCD: Thermal conductivity detector
TGA: Thermogravimetric analysis
ThOD: Theoretical oxygen demand
UHPLC: Ultra-high-performance liquid chromatography
VFs: Virulence factors
WCA: Water contact angle
WGS: Whole genome sequencing
XPS: X-ray photoelectron spectroscopy
XRD: X-ray diffraction
μ-ECD: Micro electron catching detector
